# Der Chirurg im Spagat – Lehre im klinischen Alltag

**DOI:** 10.1007/s00104-021-01470-1

**Published:** 2021-07-23

**Authors:** J. Sterz, V. Britz, P. Carstensen, T. Kollewe, S. H. Voß, M. C. Stefanescu, T. Schreckenbach, R. D. Verboket, Miriam Rüsseler

**Affiliations:** 1 Klinik für Unfall‑, Hand- und Wiederherstellungschirurgie, Universitätsklink Frankfurt, Goethe-Universität, Theodor-Stern-Kai 7, 60590 Frankfurt, Deutschland; 2 Frankfurter Interdisziplinären Simulationstraining, Fachbereich 16, Goethe-Universität, Frankfurt am Main, Deutschland; 3 Frankfurter Arbeitsstelle für Medizindidaktik, Fachbereich Medizin, Goethe-Universität, Frankfurt am Main, Deutschland; 4 MVZ VOSS, Aschaffenburg, Deutschland; 5 Klinik für Allgemein- und Viszeralchirurgie, Universitätsklinikum Frankfurt, Goethe-Universität, Frankfurt am Main, Deutschland

**Keywords:** Lehre im klinischen Alltag, Personalmangel, Nachwuchsmangel, Anerkennung, Qualitative Analyse, Teaching in daily surgical routine, Personnel shortage, Shortage of recruits, Recognition, Qualitative analysis

## Abstract

**Hintergrund:**

Aufgrund des Nachwuchsmangels muss die Chirurgie vermehrt für die Weiterbildung zum Chirurgen begeistern. Studierende bemängeln, dass ihr Unterricht nachrangig zur Patientenversorgung und durch die Faktoren Zeit und ärztliches Personal limitiert ist. Obwohl es viele Arbeiten mit Fokus auf die Studierenden gibt, fehlen Arbeiten mit dem Fokus auf die Sicht der Lehrenden.

**Ziel der Arbeit:**

Die Lehre im Fach Chirurgie im Stationsalltag und Ursachen von Problemen hierbei sollen aus Sicht der Lehrenden analysiert werden.

**Material und Methoden:**

Im Rahmen der prospektiven Studie wurde ein Leitfaden für semistrukturierte Interviews mit ausformulierten, offenen Fragen erstellt, die mit weiteren Spezifizierungsfragen versehen wurden.

Alle Interviews wurden anhand des Leitfadens geführt und aufgezeichnet. Die Anzahl der Interviews ergab sich aus dem Prinzip der inhaltlichen Sättigung.

**Ergebnisse:**

Alle der 22 befragten Ärzte messen der Lehre im klinischen Alltag einen hohen Stellenwert bei. Dennoch beschreiben sie, dass die Lernziele im klinischen Alltag nicht immer erreicht werden. Als Hauptgrund hierfür wird die mangelnde Zeit genannt. Mit zunehmender klinischer Erfahrung kommen jedoch weitere Faktoren hinzu: Ober- und Chefärzte beklagen die zu geringen Vorkenntnisse und die zu geringe Motivation einiger Studierender. Die meisten Befragten beschreiben, keine Anerkennung für ihre Lehre zu erhalten. Insgesamt wird die studentische Lehre als zusätzliche Belastung wahrgenommen, die aber alle Befragten für lohnenswert halten.

**Diskussion:**

Neben Personalmangel ist die fehlende Anerkennung das wichtigste Hemmnis für eine effektive Lehre. Es ist daher wichtig, die Wertigkeit der Lehre durch die Belohnung guter Lehrleistungen und Schaffung einer dahingehenden Transparenz zu erhöhen.

## Abstract (en)

**Background:**

Thus medical students must be inspired to undertake this specialty. Students complain that the teaching is subordinate to patient care and limited by a lack of time and medical personnel. Although there are many studies assessing student perceptions, few exist that focus on the issues that teachers face.

**Objective:**

To analyse student teaching in the daily routine and its potential’ problems from the surgeon’s perspectives.

**Material and methods:**

In this prospective study guidelines for semistructured interviews with formulated, open questions were created, which were specified with further questions. All interviews were conducted using these guidelines and recorded. The number of interviews were a function of the concept of content saturation.

**Results:**

All 22 participants perceived that the teaching in clinical practice is of paramount importance. Nevertheless, respondents described that learning goals in the clinical routine are not always achieved. The main reason is a lack of time; however, as clinical experience increases other factors will similarly become more important: Consultants and heads of departments complain about deficiencies in students’ previous knowledge, including insufficient motivation.

Most respondents described that they do not feel appreciated for teaching. Overall, student teaching was perceived as an additional burden but all respondents found the task to be extremely worthwhile.

**Conclusion:**

In addition to the lack of personnel, a lack of appreciation is the most significant obstacle towards effective teaching. It is therefore important to increase the value of teaching by rewarding good achievements and the creation of effective transparency.

Im Jahr 2030 werden schätzungsweise 7300 Chirurgen und Orthopäden im stationären Sektor fehlen. Um dem entgegenzuwirken, muss die Chirurgie vermehrt für ihr Fach werben und Studierende begeistern. Die beste Möglichkeit hierfür bietet der direkte Einblick in das Fach, den Studierende während ihrer Praktika erhalten. Aus Sicht der Studierenden bestehen hier aber große Defizite. Analysen aus Sicht der Lehrenden fehlen aber bisher. Daher ist es nötig, diese zu befragen, um Hemmnisse für eine gute Lehre im klinischen Alltag zu analysieren und Verbesserungsmöglichkeiten zu erarbeiten.

## Hintergrund

Der Nachwuchsmangel stellt die Chirurgie vor bisher nicht gekannte Probleme [20]: Im Jahr 2030 werden 7300 Chirurgen und Orthopäden im stationären Sektor fehlen [11]. Diese Entwicklung ist schon im Medizinstudium sichtbar: Sowohl bundesweite [16] Umfragen als auch Befragungen an einzelnen Fakultäten [21] zeigten, dass die Anzahl derjenigen Studierenden, die eine Weiterbildung in einem chirurgischen Fach anstreben, während des Studiums deutlich sinkt. Damit ist die Chirurgie nach der Labormedizin das Fach, das den größten Interessensverlust während des Studiums erleidet [16]. Insgesamt entschieden sich gemäß des bayerischen Absolventenpanels ein Jahr nach Studienabschluss weniger als 10 % der Absolventen für eine Weiterbildung in den chirurgischen Fächern [5].

Vor diesem Hintergrund muss die Chirurgie vermehrt für ihr Fach werben und die Studierenden für die Weiterbildung zum Chirurgen begeistern [20]. Die beste Möglichkeit bieten Blockpraktika und Famulaturen [19], da hier das Interesse nicht nur für eine PJ(Praktisches-Jahr)-Rotation in ein chirurgisches Fach, sondern auch für eine chirurgische Weiterbildung signifikant gesteigert werden kann [8, 13].

Insgesamt bewerten die Studierenden die Lehre am Krankenbett als dringend notwendig zum Erlernen praktischer Fertigkeiten [17–19, 26]. Jedoch bemängeln sie, dass ihr eigener Unterricht der Patientenversorgung nachgeordnetund damit durch die Faktoren Zeit und ärztliches Personal deutlich und zunehmend limitiert ist [19, 22, 26]. Dies konnte in verschiedenen Arbeiten bestätigt werden, die einen Rückgang der Lehre am Krankenbett beschreiben [1, 17, 18].

Hierfür gibt es multifaktorielle Gründe, insbesondere strukturelle Änderungen, die zu einer zunehmenden Arbeitsverdichtung führen wie die Verkürzung der Liegedauer mit höherem Patientenaufkommen [17] und zunehmendem Dokumentationsaufwand ohne zusätzlich geschaffene Zeiträume [1, 18]. Darüber hinaus werden vor allem die gestiegenen Anforderungen auf Seiten der Lernenden [1] und fehlendes Feedback zur Qualität der Lehre [1] sowie insbesondere die mangelnde Wertschätzung für das Engagement in der Lehre [1, 14] als wichtige Gründe diskutiert. Dies führt dazu, dass Lehre im klinischen Alltag zu selten erfolgt und gute Gelegenheiten hierfür verpasst werden [26].

Die Studierenden sind davon überzeugt, dass jeder Arzt am Krankenbett unterrichten kann [26]. Kasch et al. konnten zeigen, dass fast die Hälfte der befragten Studierenden jedoch unzufrieden mit der didaktischen Qualität der Lehre in ihrer chirurgischen Famulatur war und nur 35,3 % das stattgehabte „bedside teaching“ als gut empfanden [7].

Es existieren bisher nur wenige Arbeiten mit dem Fokus auf die Lehrenden in den Universitätskliniken. Unseres Wissens gibt es bisher sogar nur eine Arbeit, die sich mit der Lehre in den peripheren Lehrkrankenhäusern beschäftigt [15], obwohl hier der Großteil der Studierenden am Krankenbett unterrichtet wird. Ziel der vorliegenden Arbeit ist es daher, die Lehre an den Lehrkrankenhäusern und den Universitätskliniken im Fach Chirurgie im Stationsalltag aus Sicht der Lehrenden genauer zu beleuchten und Ursachen möglicher Probleme und Hemmnisse aufzuzeigen.

## Material und Methoden

Das Studiendesign war prospektiv. Gemäß Aussage der Ethikkommission der Universitätsklinik Frankfurt war für die vorliegende Studie kein Ethikvotum notwendig, da es sich nicht um ein biomedizinisches Forschungsvorhaben im engeren Sinne der Deklaration von Helsinki handelt. Die Teilnahme an der Studie erfolgte freiwillig mit schriftlicher Einverständniserklärung und konnte jederzeit ohne Angabe von Gründen beendet werden.

### Fragebogenerstellung

Basierend auf den Fragestellungen der Studie wurde ein Leitfaden für semistrukturierte Interviews erstellt. Der Leitfaden diente dazu, die Interviewthematik einzugrenzen und Themenkomplexe vorzugeben, um die Vergleichbarkeit der Ergebnisse der verschiedenen Interviews zu sichern. Der Leitfaden wurde in einem Probeinterview mit Chirurgen, die nicht an der Erstellung des Leitfadens beteiligt waren, getestet und basierend hierauf erneut verfeinert. Nach der Überarbeitung bestand der Leitfaden aus 15 offenen, ausformulierten Fragen, von denen jede mit weiteren Spezifizierungsfragen versehen war. So stellte der Leitfaden die Vergleichbarkeit der einzelnen Interviews sicher.

### Durchführung der Interviews

Die Rekrutierung der Teilnehmer erfolgte durch direkte Ansprache der Lehrenden an der Universitätsklinik Frankfurt und an den akademischen Lehrkrankenhäusern. Die Teilnahme erfolgte freiwillig nach einer ausführlichen Aufklärung über die Studie und die damit verbundene Tonaufnahme sowie der Einwilligung zur anonymisierten Auswertung und zur späteren Veröffentlichung der Studienergebnisse. Eine Aufwandsentschädigung erhielten die Teilnehmer nicht.

Alle Interviews erfolgten in einer reizarmen Umgebung, wobei nur der interviewte Dozent und der Interviewer anwesend waren. Die Interviews wurden mittels eines Tonaufnahmegerätes aufgezeichnet. Jedes Interview wurde mit einer standardisierten Eingangsfrage eröffnet. Im Verlauf des Interviews wurde die Reihenfolge der Fragen in Abhängigkeit vom Gesprächsverlauf variiert. Die Interviews endeten, wenn alle Fragen zur Zufriedenheit der Dozenten beantwortet waren. Die Anzahl der geführten Interviews ergab sich aus dem Prinzip der inhaltlichen Sättigung.

### Datenanalyse und Auswertung

Alle Interviews wurden wortwörtlich unter Verwendung des Programms f5 (dr. dresing & pehl GmbH, Marburg, Deutschland) transkribiert und zur weiteren Auswertung anonymisiert. Die Auswertung erfolgte mit dem Programm MAXQDA (VERBI Software, Consult Sozialforschung GmbH, Berlin, Deutschland) mittels strukturierender Inhaltsanalyse [12]. Hierfür erfolgte zunächst basierend auf dem Leitfaden die Definition von Hauptkategorien und ersten Unterkategorien, anhand derer alle Interviews durch zwei der Autoren unabhängig voneinander ausgewertet wurden. Im Anschluss wurden abweichende Codierungen diskutiert und die Definitionen der Kategorien verfeinert. Anhand dieser Definitionen wurden alle Interviews ausgewertet.

## Ergebnisse

Insgesamt wurden 22 Interviews geführt. Die soziodemographischen Daten der Teilnehmer sind in Tab. 1 dargestellt.

**Table 1:** 

	Gesamt	Frauen	Männer
Teilnehmer	22	6	16
*Chirurgisches Fach*			
Allgemeinchirurgie	8	2	6
Gefäßchirurgie	2	0	2
Herz- und Thoraxchirurgie	3	0	3
Mund‑, Kiefer- und plastische Gesichtschirurgie	4	0	4
Unfallchirurgie	5	4	1
*Weiterbildungsstand*			
Chefarzt	3	0	3
Oberarzt	7	1	6
Facharzt	1	0	1
Assistenzarzt	11	5	6
*Arbeitsstätte*			
Universitätsklinik	14	5	9
Akademisches Lehrkrankenhaus	8	1	7

Alle im Rahmen der vorliegenden Arbeit befragten Ärzte (22/22) messen der Lehre im klinischen Alltag einen hohen Stellenwert bei. Die Bedeutung der Ausbildung des Nachwuchses für die Zukunft ist allen Teilnehmenden bewusst. Die Wichtigkeit, die die Befragten der Lehre im klinischen Alltag beimessen, ist hierbei unabhängig vom Weiterbildungsstand.

Also ich finde es super wichtig, weil man ja Ärzte von morgen ausbildet und wenn die es nicht mitbekommen im Studium, wann sollen sie es denn dann lernen?

Ich nehme das relativ ernst, weil ich der Meinung bin, dass eben auch kommende Generationen von Patienten vor allem davon profitieren, wenn wir als Jungmediziner den noch Jüngeren oder Studenten Sachen beibringen.

Eine große Bedeutung kommt in der Lehre den Lernzielen zu. Dennoch beschreiben die Befragten, dass die Lernziele, die sie für die Studierenden im klinischen Alltag definieren, nicht immer erreicht würden. Hierfür werden verschiedene Gründe angeführt (siehe auch Tab. 2). Unabhängig sowohl vom eigenen Stand der Weiterbildung als auch von der Art der Arbeitsstätte wird der Zeitmangel als Haupthemmnis für Lehre im klinischen Alltag angeführt:

Zeit. Wir sind ja so straff eingebunden, dass wir uns auch ohne Studenten nicht langweilen, und wenn wir dann die Aufgabe zusätzlich noch kriegen, den Studenten adäquat zu unterrichten, geht das dann von meiner Freizeit ab.

**Table 2:** 

Hemmnis	Anzahl der Nennungen
Zeit- und Personalmangel	19 (86,4 %)
Zu geringe Motivation der Studierenden	10 (45,4 %)
Unangemessene Erwartungen der Studierenden	3 (13,6 %)
Mangelhafte Vorkenntnisse der Studierenden	2 (9,1 %)

Mit zunehmender klinischer Erfahrung der Dozenten kommen jedoch weitere Faktoren hinzu: Besonders die von uns befragten Ober- und Chefärzte beklagen teilweise eklatante Mängel bezüglich der Vorkenntnisse der Studierenden, welche ein effektives Unterrichten im klinischen Alltag nicht möglich machen. Beklagt wird hierbei, dass ohne fundierte theoretische Kenntnisse, welche die Studierenden im Vorfeld des Stationseinsatzes beispielsweise in Vorlesungen und Seminaren erworben haben müssen, keine effektive Lehre auf der Station möglich sei.

Aber es fängt schon allein damit an, dass die fundierten theoretischen Kenntnisse erstmal vorhanden sein müssen...

Weil die mit zum Teil extrem unterschiedlichen Vorkenntnissen kommen und um es mal etwas provokant zu sagen, die Vorkenntnisse sind relativ gering [...] Wenn man die Verkehrsregeln nicht kennt, kann man nicht Autofahren. Wenn man keine Ahnung von Chirurgie hat, ist man hier [...] interessierter Laie.

Darüber hinaus sehen die durch uns befragten Chirurgen ein weiteres Hemmnis für effektive Lehre im klinischen Alltag in der ihrer Meinung nach teilweise zu geringen Motivation einiger Studierender. Dies werde insbesondere durch das Pflichttertial Chirurgie begünstigt, da hier nicht nur Studierende unterrichtet werden müssen, die ein originäres Interesse am Fach haben, sondern eben auch solche, die bereits wissen, dass sie nicht in der Chirurgie arbeiten wollen.

Und es gibt immer mal Probleme, wenn dann Studenten dazwischen sind, die völlig desinteressiert sind.

Es gibt Leute, die halt mit Desinteresse ankommen, wo man keinen Spaß hat, denen was beizubringen, [...] bin ich froh, wenn die nach ‘ner Zeit wieder gehen.

Die befragten Ober- und Chefärzte berichten zudem, dass mangelnde Vorkenntnis und eine zu geringe Motivation noch dadurch verschärft würden, dass die Studierenden häufig mit nicht ihrem Ausbildungsstand angemessenen Erwartungen in die Klinikeinsätze starten würden. Durch diese erhöhte Erwartung käme es zu Frustration auf beiden Seiten.

Dass gar nicht mehr so die Bereitschaft da ist, was beigebracht zu bekommen, weil’s halt ihren Erwartungen nicht entspricht. So von wegen: „Wie Verbandswechsel? Ich will Arzt werden, das macht eine Schwester“.

Die heutigen Studenten verlangen viel mehr. Und wissen gar nicht, in Anführungsstrichen, was auf den Arzt täglich zukommt

Zusätzlich beschreiben die Ober- und Chefärzte auch Einschränkungen aufgrund der organisatorischen Rahmenbedingungen: Als besonders limitierend empfinden sie, dass häufig auf den Stationen gerade diejenigen für die Betreuung der Studierenden zuständig seien, die selbst noch unerfahren sind. Hierdurch käme es zu der Situation, dass die jüngsten ärztlichen Kollegen, während sie selbst noch ein Gefühl der Überforderung erleben und für die Ausführung ihrer Aufgaben länger benötigen als die erfahreneren Kollegen, zusätzlich zu ihren Aufgaben in der Patientenversorgung auf der Station gleichzeitig noch für die Betreuung der Studierenden verantwortlich seien. Ihnen fehle nicht nur die Zeit, sondern auch das nötige Wissen, um die Studierenden unterrichten zu können.

... mit einem jungen Assistenzarzt, der selber noch nicht weiß, wo die Herrentoilette ist, sagen wir mal, ist das halt ein Problem.

… dass man den Jüngsten die Verantwortung für die Studierenden überträgt und das als selbstverständlich erachtet, dass genau die das auch können sollen. [...] Die wissen mit sich selbst nichts anzufangen. Die brauchen selbst noch Hilfe bei der Stationsführung und da kann man nicht noch sagen, den bildest du jetzt noch mit aus.

Nur zwei der von uns befragten Dozenten (9,1 %) geben an, regelmäßig und strukturiert Feedback für ihre Lehrleistung zu erhalten. Dennoch beschreiben die in der vorliegenden Studie befragten Dozenten, dass das häufig positive Feedback der Studierenden für sie die wichtigste Form der Anerkennung für ihre Bemühungen um die studentische Lehre sei. Sie fühlten sich hierdurch in ihren Bemühungen bestärkt und seien daher auch gerne bereit, Zeit in die Lehre zu investieren, auch wenn sie dafür Überstunden machen müssen.

Ich krieg schon Anerkennungen, insofern, dass ich glaube, dass Studenten die gefördert werden, auch sehr dankbar sind.

Ja, ein Benefit ist natürlich darin zu sehen, dass man Leute für den eigenen Beruf interessiert.

Ob die Dozenten darüber hinaus eine weitere Anerkennung von Seiten ihrer Vorgesetzten erhalten, wird von ihnen sehr unterschiedlich wahrgenommen. Insgesamt beschreiben die meisten Befragten, keine Anerkennung für ihre Lehre zu erhalten (68,2 % oder 15/22). Diejenigen Dozenten, die angeben, dass ihre Tätigkeit in der Lehre anerkannt wird, erfahren diese Anerkennung meist von ihren Vorgesetzten, weniger von Seiten der Klinikverwaltung. Auffällig hierbei ist, dass es keinen Zusammenhang zwischen der wahrgenommenen Anerkennung der Lehrtätigkeit und der Art der Arbeitsstätte gibt.

Da krieg ich kein Dankeschön, vielleicht von den Studenten, ich krieg eher Druck, die Klinik geht vor und dann macht man ‘ne Triage und sagt, okay, jetzt muss ich abwägen.

Eine Anerkennung von Seiten des Arbeitsgebers bekomme ich nicht. Ich bekomme eine Anerkennung von Seiten meines Chefs, der sehr wohl feststellt [...], dass die Studenten hier zufrieden sind.

Nur 27,3 % (6/22) der befragten Dozenten geben an, didaktisch ausreichend geschult zu sein. Diejenigen Befragten, die sich nicht ausreichend geschult fühlen, empfinden dies als nachteilig.

Nein. Gar nicht. Niemals im Studium und auch später nicht.

Relativ schockierend wenig, würde ich sagen.

Die Dozenten, die sich als ausreichend geschult betrachten, sind hauptsächlich diejenigen, die bereits im Rahmen eines Habilitationsverfahrens Didaktikkurse besucht haben. Diese Kurse werden von den Befragten, die sie bereits besucht haben, als sehr hilfreich bewertet. Auch die jüngeren Dozenten schätzen den Besuch von Didaktikkursen als prinzipiell hilfreich ein. Sie berichteten aber, dass sie häufig keine Möglichkeit sähen, hierfür in ihrem ohnehin vollen Zeitplan Freiräume zu schaffen.

Sachen, an die man vorher im Prinzip nicht gedacht hat, weil die didaktische Aus- und Weiterbildung in der Medizin im Prinzip überhaupt nicht vorkommt und so kann man denke ich aus jedem dieser Kurse was ziehen.

…dass die Jobkomponente so schon genug Herausforderungen und zeitliche Einnahme darstellt. Ich würd’, glaube ich, davon mit Sicherheit profitieren, wüsste aber nicht, wo ich das in meiner Freizeit noch unterbringen kann ehrlicherweise.

Vor diesen Hintergründen wird die studentische Lehre auf den chirurgischen Stationen generell als zusätzliche Belastung wahrgenommen. Alle von uns befragten Dozenten betonten aber, diese Belastung als gewinnbringend und lohnenswert zu empfinden.

Es macht auch Spaß und dafür bleibt man ehrlich gesagt auch gerne mal eine viertel oder halbe Stunde länger. Ist schon ok.

Eine zusätzliche Belastung ist es, zweifelsohne. Aber es ist es wert.

## Diskussion

Die vorliegende Arbeit belegt, dass die Lehre im klinischen Alltag für Chirurgen sowohl an den Universitätskliniken als auch an den akademischen Lehrkrankenhäusern einen hohen Stellenwert hat. Dennoch zeigt sie auch, mit welchen teilweise eklatanten Hemmnissen die Lehrenden konfrontiert sind, insbesondere dem Mangel an Zeit zum Lehren. In ihrem Review aus dem Jahre 2014 zeigten Peters und ten Cate ähnliche Ergebnisse auf: Ein häufiger Grund für den Rückgang des „bedside teaching“ sind die Veränderungen, mit denen die Krankenhäuser in den letzten Jahren konfrontiert waren, wie z. B. die Verkürzung der Liegedauer oder die zunehmende Arbeitsbelastung der Ärzte [17]. Auch für Deutschland sind eine zunehmende Arbeitsplatzbelastung, gestiegene Patientenzahlen und eine Zunahme der patientenfernen Tätigkeiten belegt [2]. Hier bedarf es einer verbesserten Unterstützung der Lehrenden auf der Station mit der Schaffung von Zeiträumen, die der Lehre vorbehalten sind und nicht durch Überstunden kompensiert werden müssen. Dies wird mit dem Eintritt der Generation Y in den Arbeitsmarkt umso bedeutsamer, da für diese Generation eine ausgeglichene Work-Life-Balance einer der wichtigsten Faktoren für die Wahl des Arbeitsplatzes ist [7]. Auch für andere klinische Fächer kann angenommen werden, dass Herausforderungen wie die Verkürzung der Liegezeit oder die zunehmende Arbeitsbelastung Hemmnisse für die Lehre im klinischen Alltag darstellen. Aussagekräftige Studien hierzu fehlen allerdings bisher. Weitere Studien sollten daher darauf fokussieren, welche der in der vorliegenden Studie aufgezeigten Hindernisse auch in anderen klinischen Fächern relevant sind und ob es Unterschiede in den wahrgenommenen Belastungen zwischen den Fächern gibt.

Die in der vorliegenden Arbeit befragten Dozenten berichten, im klinischen Alltag zu wenig Zeit für die Lehre der Studierenden zur Verfügung zu haben. Die Studierenden befürchten häufig bereits vor ihrem tatsächlichen Stationseinsatz, dass sie zu wenig ärztliche Betreuung erfahren werden [10]. Dies bestätigt sich insbesondere im Praktischen Jahr, hier beschreiben die Studierenden zu wenig Zeit und zu wenig Supervision durch die sie betreuenden Ärzte, sie plädieren daher für die Einrichtung von ausdrücklich für die Lehre freigehaltenen Zeitfenstern [22]. Auch in einer bundesweiten Umfrage unter Medizinstudierenden im chirurgischen Tertial des Praktischen Jahres bestätigte sich dies: Nur 38,3 % der Studierenden bewerteten den Kontakt zu den Lehrenden als gut oder sehr gut. Hierbei zeigten sich diejenigen Studierenden zufriedener, die über einen guten oder sehr guten Kontakt zu den betreuenden Ober- und Fachärzten berichteten [4]. Dieses Manko erscheint umso gravierender, da Meder et al. belegen konnten, dass mit intensiver, qualitativ hochwertiger Lehre Studierende für ein chirurgisches Fach begeistert werden können [13]. Da die Chirurgie wie wenige andere Fächer mit einem massiven Nachwuchsmangel konfrontiert ist [20, 24, 25], erscheint es umso notwendiger, die Studierenden durch hochwertige Lehre für eine spätere Tätigkeit in einem chirurgischen Fach zu begeistern.

In der vorliegenden Arbeit gaben nur 27,3 % der Dozenten an, ausreichend didaktisch für die Lehre im klinischen Alltag geschult zu sein. Diese subjektive Wahrnehmung der Dozenten spiegelt sich auch in der Bewertung durch die Studierenden wider: Fröhlich et al. zeigten, dass die didaktische Qualität der Lehre nur von 37,8 % der Studierenden im Pflichttertial Chirurgie als gut bis sehr gut bewertet wird [4]. Eine fundierte Ausbildung der Studierenden im klinischen Alltag ist aber nur möglich, wenn die Dozenten ausreichend didaktisch geschult sind. Dies wird in den kommenden Jahren umso wichtiger, da im Zuge des Masterplanes 2020 umfassende Änderungen nicht nur für das Medizinstudium als solches, sondern auch eine grundlegende Umgestaltung des M3-Examens geplant sind [3, 6]. Hierbei sollen vermehrt diejenigen Kompetenzen geprüft werden, die die Studierenden im direkten Umgang mit den Patienten und Kollegen auf den Stationen erwerben müssen. Gemeinsam mit den Ergebnissen der vorliegenden Studie unterstützt dies die Forderung von Stieger et al. nach der Etablierung klinischer Lehrexperten, die sowohl die fachärztliche Qualifikation als auch die medizindidaktische in sich vereinen und so neben der klinischen Lehre auch Aufgaben in Kurrikulums- und Prüfungsentwicklung, Koordination und Organisation der Lehre sowie evidenzbasierter Lehr- und Lernforschung erfüllen sollen [23]. Doch auch für die Qualifikation zu einem solchen klinischen Lehrexperten muss durch die Dozenten zusätzliche Zeit aufgebracht werden. In der vorliegenden Studie berichten besonders die noch unerfahreneren Dozenten, diese Zeit für ihre eigene didaktische Weiterbildung nicht mit dem ohnehin vollen Dienstplan vereinbaren zu können. Hier erscheint die Förderung von mit dem klinischen Alltag vergleichbaren Kursen, wie beispielsweise dem durch die chirurgische Arbeitsgemeinschaft Lehre der Deutschen Gesellschaft für Chirurgie initiierten Train-the-Trainer-Kurs, unbedingt erforderlich [1].

Insgesamt betrachten alle in der vorliegenden Arbeit befragten Dozenten die Lehre im klinischen Alltag als lohnenswert. Als starker Motivator wird hierbei das positive Feedback durch die Studierenden genannt, während Anerkennung oder Feedback durch die Vorgesetzten oder die Kliniken selbst häufig fehlen. Hierin unterscheiden sich die chirurgischen Dozenten nicht von anderen Hochschullehrern. Kiefer et al. zeigten 2013, dass, obwohl die Lehre an sich 97 % der Hochschullehrer Freude bereitet, 53 % in dieser Hinsicht grundsätzlichen Veränderungsbedarf sehen. Nur 37 % der Hochschullehrer berichten, Wertschätzung für gute Lehre durch ihre Vorgesetzten zu erhalten [9]. Als Hauptmotivator wird auch hier der Wunsch, die Studierenden für das eigene Fach zu begeistern, und das positive Feedback durch die Studierenden berichtet [9]. Insgesamt erscheint es daher notwendig, standardisierte Bewertungsmöglichkeiten für die Lehre im klinischen Alltag zu schaffen, durch die niederschwellig und transparent eine Anerkennung guter Lehrleistung durch Vorgesetzte und Kliniken begründet werden kann.

## Schlussfolgerung

Neben Personalmangel und dem daraus resultierende Mangel an Zeit erscheint die fehlende Anerkennung der Lehre im klinischen Alltag das wichtigste Hemmnis für eine effektive Lehre. Somit erscheint es wichtig, neben einer nachhaltigen Verbesserung der personellen Rahmenbedingungen, die Wertigkeit der Lehre durch die Wertschätzung oder Belohnung guter Lehrleistungen und Schaffung einer dahingehenden Transparenz zu erhöhen. So können durch qualitativ hochwertige Lehre Studierende für die Chirurgie begeistert werden.

## Fazit für die Praxis

- Obwohl Chirurgen die Lehre als absolut lohnenswert empfinden, betrachten sie sie als zusätzliche Belastung.
- Neben Personal- und Zeitmangel ist fehlende Anerkennung der Lehre im klinischen Alltag das wichtigste Hemmnis für eine effektive Lehre.
- Es bedarf einer verbesserten Unterstützung der Lehrenden auf der Station mit der Schaffung von Zeiträumen, die der Lehre vorbehalten sind und nicht durch Überstunden kompensiert werden müssen.
- Die Wertigkeit der Lehre sollte durch eine vermehrte Wertschätzung oder Belohnung guter Lehrleistung durch die Kliniken erhöht werden.
